# Galaxy Integrated Omics: Web-based Standards-Compliant Workflows for Proteomics Informed by Transcriptomics*[Fn FN2]

**DOI:** 10.1074/mcp.O115.048777

**Published:** 2015-08-12

**Authors:** Jun Fan, Shyamasree Saha, Gary Barker, Kate J. Heesom, Fawaz Ghali, Andrew R. Jones, David A. Matthews, Conrad Bessant

**Affiliations:** From the ‡School of Biological and Chemical Sciences, Queen Mary University of London, Mile End Road, London E1 4NS, UK;; §School of Cellular and Molecular Medicine, University of Bristol, University Walk, Bristol. BS8 1TD, UK;; ¶School of Biological Sciences, University of Bristol, University Walk, Bristol. BS8 1TD. UK.;; ‖School of Biochemistry, University of Bristol, University Walk, Bristol. BS8 1TD, UK;; **Institute of Integrative Biology, University of Liverpool, Liverpool, L69 7ZB, UK

## Abstract

With the recent advent of RNA-seq technology the proteomics community has begun to generate sample-specific protein databases for peptide and protein identification, an approach we call proteomics informed by transcriptomics (PIT). This approach has gained a lot of interest, particularly among researchers who work with nonmodel organisms or with particularly dynamic proteomes such as those observed in developmental biology and host-pathogen studies. PIT has been shown to improve coverage of known proteins, and to reveal potential novel gene products. However, many groups are impeded in their use of PIT by the complexity of the required data analysis. Necessarily, this analysis requires complex integration of a number of different software tools from at least two different communities, and because PIT has a range of biological applications a single software pipeline is not suitable for all use cases. To overcome these problems, we have created GIO, a software system that uses the well-established Galaxy platform to make PIT analysis available to the typical bench scientist via a simple web interface. Within GIO we provide workflows for four common use cases: a standard search against a reference proteome; PIT protein identification without a reference genome; PIT protein identification using a genome guide; and PIT genome annotation. These workflows comprise individual tools that can be reconfigured and rearranged within the web interface to create new workflows to support additional use cases.

Searching MS/MS spectra against a list of proteins that could be present in the sample remains the pre-eminent method for identifying proteins in liquid chromatography tandem mass spectrometry (LC-MS/MS)^1^ shotgun proteomics. The relevance and quality of this *protein database*, as it is known, has been shown to have a significant impact on the outcome of a proteomics study (1), but even for well-studied model organisms the true set of proteins that might be expressed is debatable. For example, large scale human proteome mapping projects have recently suggested that commonly used human protein databases may include superfluous proteins while simultaneously omitting proteins for which there is evidence of expression (2, 3). For lesser studied organisms the situation is worse still, as available protein databases rely even more on computational prediction, and in some species even this is not possible as a reference genome has not yet been assembled. This is a significant problem in fields such as virology, where the disease vectors (*e.g.* mosquitoes, lice, ticks, birds, and bats) have poorly annotated genomes or no genome data at all. Similar challenges can be found in metaproteomics (4), where individual samples contain proteins from multiple organisms, some of which may be unidentified or not previously sequenced.

Recently, we sought to mitigate these problems by developing a methodology called proteomics informed by transcriptomics (PIT) (5) in which sample-specific protein databases are generated from transcripts that have been identified in the same sample using RNA-seq (6). These transcripts can be assembled from short reads either by mapping to a genome or entirely *de novo* (*e.g.* if no suitable genome exists). Although we showed that PIT could provide valuable new insights into the systems being studied, the complexity of the software pipeline needed to integrate the RNA-seq and proteomic MS/MS data made it difficult to configure the method for different applications and to adapt it to make use of new transcriptomic and proteomic tools. In this paper we introduce our solution to these challenges—a publicly available standards-compatible system called GIO (Galaxy Integrated Omics) that uses the popular Galaxy platform (7) to make flexible PIT workflows available via an easy to use web interface. We explain the workflows, the GIO implementation of these workflows and validate the efficacy of this software using a matched RNA-seq and LC-MS/MS dataset.

## EXPERIMENTAL PROCEDURES

### 

#### 

##### Software Implementation

##### Use of the Galaxy Platform

Galaxy (7) is a highly customizable server-based bioinformatics platform that has already amassed a large following among the genomics community as a framework within which complex analysis of large data sets can be easily conducted in a repeatable way by non-bioinformaticians. It provides a powerful web interface through which data can be uploaded, tools executed, and workflows built, inspected, executed, and shared. Because all the necessary tools and workflows reside on a centrally managed server the need for users to download, install, and configure software is avoided. This makes Galaxy an obvious platform for PIT because PIT requires integration of a wide range of individual tools from different domains. The simple concept behind Galaxy is that any command line tool can be added to it by writing a *wrapper* that lists the tool's parameters, specifies its inputs and outputs, and defines the command line syntax used to run the tool. Galaxy uses this wrapper to automatically create a user interface for each tool, substituting the tool's command line interface with a simple web-based form. Because they define each tool's input and output formats, wrappers also allow tools to be linked together into workflows. Wrappers allow a tool to be incorporated into Galaxy without changing the tool's program code, regardless of the programming language in which the tool was written. Although it is possible to make tools and workflows available for administrators of Galaxy servers to download and install via a repository called Galaxy Tool Shed, configuring all the necessary components can be complicated so we chose instead to provide our workflows via a dedicated customized version of Galaxy containing all relevant tools and workflows. A demonstration of this customized Galaxy version, which we call Galaxy Integrated Omics (GIO), is freely accessible at gio.sbcs.qmul.ac.uk. We also maintain a repository of the core GIO components, together with an automated installation program, on GitHub (https://github.com/wizardfan/gio-repository) so that groups who wish to add GIO functionality to their own Galaxy server can do so.

##### Choice of Tools

There is a large and growing range of existing command line proteomics tools available, offering a range of functionality under various licensing arrangements (*e.g.* protein quantitation tools have recently been reviewed (8)). For GIO we have selected a subset of tools that covers the steps needed to construct PIT workflows. These tools were chosen based on their perception within the community and their ability to support data standards (see next section). The key transcriptomic tools were already available in Galaxy Tool Shed, as were some of the proteomics tools (wrapped by the GalaxyP project (9)). Given the difficulties of definitively benchmarking the efficacy of individual tools it is impossible to be certain that our selection is optimal, but the modular nature of Galaxy makes it straightforward to add new tools, to replace individual workflow steps in the future as better tools become available. Because the PIT workflows contain novel functionality it was necessary to implement a number of new tools, which are distinguished in GIO by names prefixed with “PIT:.” These tools include a bespoke ORF finder called PIT:ORFall, and PIT:Integrate for bringing transcriptomic and proteomic data together using our previously described method (5). Some of the PIT tools are themselves mini-workflows that combine workflow steps that are commonly used together. For example, PIT:PSM PostProcessing sequentially runs three individual tools from mzIdentML-lib (10) to determine PSM level FDR scores, remove peptides below a user-defined threshold, and map peptides to proteins. Such meta-tools simplify the creation of workflows, improving their readability and reducing the chance of errors.

##### Data Standards

One of the prerequisites for building workflows is that individual tools must be compatible such that the output of one tool can be used as the input to another. To ensure this, GIO supports PSI data standards (10). In particular, we provide a number of tools from mzIdentML-lib (11) to support the manipulation of the mzIdentML (12) files. For spectral data, GIO supports the PSI standard mzML (13) format. Data in other formats can be converted to mzML using ProteoWizard's MSConvert (14) within GIO. Because the libraries needed to convert proprietary raw binary data formats are only available for Microsoft Windows, MSConvert has been configured to run within a Windows virtual machine on the GIO server, which is accessed by GIO using Pulsar (15).

##### Data Processing Workflows

We have developed three primary workflows for analyzing data from PIT experiments, and a standard identification workflow for comparison purposes. Each of these workflows is available to view, edit, and execute on the GIO server (gio.sbcs.qmul.ac.uk) where detailed tutorials are also provided. Applying a workflow to a given data set is simply a case of uploading and selecting which data files to use, selecting the workflow, and clicking the execute button to run the workflow. All workflow parameters (*e.g.* false discovery rate, threshold) can be set by the user, and workflows can be substantially modified or created from scratch using the available tools (*e.g.* multiple different tools are offered for peptide spectrum matching via a tool called “Peptide spectrum matching” that wraps SearchGUI (16)).

##### Standard Identification Workflow

Included primarily for testing and comparison purposes, this is a standard protein identification workflow to which the inputs are a spectra file and a list of protein sequences to search against. By default, input formats for these files are mzML and FASTA respectively, but any common formats (including proprietary .raw) can be accommodated by using conversion tools within GIO. The first step of the workflow is peptide spectrum matching, performed by MS-GF+ (17). This is followed by a post processing step that combines three tools from mzIdentML-lib (11) to calculate the false discovery rate (FDR) for each peptide, removes peptides below a specified threshold, and infers the identity of proteins from the surviving peptides. The output of these steps is a single mzIdentML file, from which the protein identifications are extracted and converted into a tabular (tab separated values - TSV) file for convenient viewing or downloading via the Galaxy interface. This workflow is shown schematically in supplemental Fig. S1 in the supplementary materials. In the common scenario where a sample has been fractionated prior to MS, the ProteoWizard MSConvert tool (14) within GIO can be used to merge all spectra data files into a single file for input to the workflow.

##### PIT Workflow without Reference Genome

**Fig. 1.**
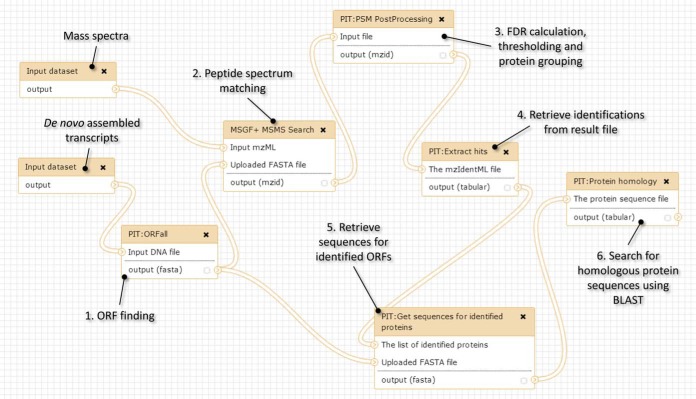
**Annotated PIT workflow without reference genome.** The inputs to the workflow are a file containing raw spectral data and a list of transcripts assembled from RNA-seq data (assembled in a separate workflow using Trinity). In the first step of analysis, the longest open reading frames (ORFs) in all six frames for each transcript are determined using the in-house ORFall tool. MS-GF+ then finds peptide spectrum matches, which are post processed and grouped to proteins using tools from mzIdentML-lib. This results in a list of confidently identified ORFs in mzIdentML format, which is then BLASTed against protein databases to add context to the results.

This is a development of the standard identification workflow in which one of the inputs is a list of *de novo* assembled transcripts instead of a protein list. To reduce load on the public GIO server, we request that the transcript list is assembled from acquired RNA-seq data outside of GIO using a stand-alone tool such as Trinity (18) and then imported into GIO, but on a locally installed GIO installation the transcript assembly step can be fully integrated into the workflow. The first step of the workflow (shown in Fig. 1) produces a protein database comprising the longest open reading frames (ORFs) found within all six reading frames of each transcript. This database is then used in the peptide spectrum matching step, which is followed by the same post processing as in the previous workflow. Because the ORFs identified from this process are unannotated sequences, further post processing is needed to infer what they may be. This takes place in the final part of the workflow, where all identified ORFs are BLASTed to find homologous proteins in selected species. The final result of the workflow is therefore a tabular file containing a list of ORFs for which peptide evidence has been found, together with an indication of the closest homologous protein in the selected species. This allows for the analysis of a sample from a species for which a reference genome is not available, by postidentification comparison with proteins in a closely related species. It also facilitates metaproteomics where proteins from two or more species (some of which may be unidentified) are present in the sample.

##### PIT Workflow for Genome Annotation

If a genome exists for the species under study, an alternative workflow can be used that includes additional steps to annotate the genome. In this workflow, in parallel with the peptide and protein identification, *de novo* assembled transcripts from the input file are mapped to the genome using GMAP (19). At the end of the workflow the in-house PIT:Integrate tool is used to integrate the identified transcriptomic and proteomic features into a single GFF3 genome annotation file so that these features can be viewed in their genomic context using a genome browser. The same information is provided in tabular and SAM formats and, as in the previous workflow, identified sequences are provided in FASTA format so a homology search can be added at the end of this workflow to provide information as to what the identified proteins may be. This workflow is shown schematically in supplemental Fig. S2 in the supplementary materials.

##### PIT with Reference Genome

If the species under study has a well annotated reference genome then instead of *de novo* transcript assembly, transcript sequences can be determined by mapping the acquired RNA-seq data to the reference genome using TopHat (20) and assembling using Cufflinks (21). This functionality is provided by a dedicated GIO workflow. In this workflow, instead of assembled transcripts, inputs are RNA-seq FASTQ files containing quality controlled forward and reverse reads. The first step in the workflow uses TopHat to map short reads to the genome of the species under study. This produces a BAM mapping file which is passed to the next step in which Cufflinks assembles the reads into transcripts. These transcripts are then used, as in the other workflows, to generate ORFs against which the acquired MS spectra are searched. Downstream processing also follows the same process as described previously for the other workflows. This workflow is shown schematically in supplemental Fig. S3 in the supplementary materials.

##### Workflow Evaluation

Workflows were evaluated by applying them to previously published (5) matched sets of RNA-seq and proteomic MS/MS data acquired from HeLa cells infected with adenovirus. This data represents a good case study because the samples contain proteins from at least two species, and the data has already been extensively analyzed using established tools so there is an opportunity to compare results. Furthermore, the human proteome is arguably the best annotated of any species, so it is possible to make a direct comparison of the results obtained with and without the use of a reference proteome.

Detailed information about the laboratory protocols can be found in the original publication (5), but in summary this was a triple SILAC experiment in which different labels represented different time points post infection. Cells were labeled with either (1) 15N- and 13C-labeled arginine and lysine (heavy HeLa), (2) 13C-labeled arginine and lysine (medium HeLa), or (3) were not labeled at all (light HeLa). The medium and light HeLa cells were infected with adenovirus, and the heavy HeLa cells were mock infected. Light cells were harvested at 8 h postinfection, and medium and heavy cells harvested after 24 h. The three samples were mixed together in a 1:1:1 ratio, digested with trypsin, and subjected to CID LC-MS/MS shotgun proteomics on a Thermo Orbitrap Velos mass spectrometer. Cytoplasmic mRNA was harvested from the individual samples and RNA-seq conducted using an Illumina GAIIx, resulting in the acquisition of ∼82 M paired-end reads, with an average length of 56 bps.

Because the purpose of this study is protein identification, peptide spectrum matching was performed with the SILAC modifications (^13^C_6_^15^N_2_-Lys and ^13^C_6_^15^N_4_-Arg for heavy labeled samples and ^13^C_6_-Lys and ^13^C_6_-Arg for medium) as variable modifications, essentially treating the data as a pooled sample. Oxidation of methionine was also included as a variable modification, and carbamidomethylation of cysteine as a fixed modification. Peptide spectrum matching was performed for fully tryptic peptides, with any reasonable number of missed cleavages (according to the MSGF+ algorithm), and mass tolerances of 10 ppm for precursor ions and 0.8 Da for product ions. A peptide level FDR threshold of 1% was applied throughout, and only proteins with at least two identified peptides were retained. All data used in this article is available as a Data Library within GIO.

## RESULTS

### 

#### 

##### Standard Identification Workflow

Searching the acquired MS/MS data against a FASTA file concatenated from the complete Uniprot proteomes of human (including isoforms) and adenovirus type 5, 26,401 peptides were found by MS-GF+, which mapped to 3,015 proteins (2997 human and 18 human adenovirus). These figures (provided in Table I), and the list of identified proteins that they represent, are comparable with those obtained from the original analysis (5) of the data using the Andromeda (22) search engine via MaxQuant. The results of this standard workflow are used below as a benchmark against which to evaluate the other workflows.

**Table I:** Summary of RNA-seq data, databases sizes, and results from de novo, genome guided PIT and standard searches

	Standard (human and adenovirus)	PIT *De novo* assembly	Standard (human only)	Genome guided assembly
Transcripts	n/a	103,431	n/a	37,024
Average transcript length	n/a	670.03	n/a	1,007.21
Total components/genes	n/a	92,661	n/a	33,747
Transcript N50	n/a	1,040	n/a	1,764
Proteins/ORFs	88,701	78,493	88,665	54,184
Identified peptides	26,401	25,907	25,970	21,601
Identified PAGs	3,015	2,926	2,986	2,713
Peptide level overlap	88%		77%	
PAG level overlap	87%		75%	

##### PIT Workflow without Reference Genome

Searching the acquired MS/MS data against ORFs derived from Trinity *de novo* assembly of RNA-seq transcripts identified 25,907 peptides, which mapped to 2926 ORFs. Using the homology search tool included in this workflow, 2889 of these ORFs were identified as human proteins, and 18 identified as adenovirus proteins. Nineteen of the ORFs had no significant homology to either human or adenovirus proteins.

**Fig. 2.**
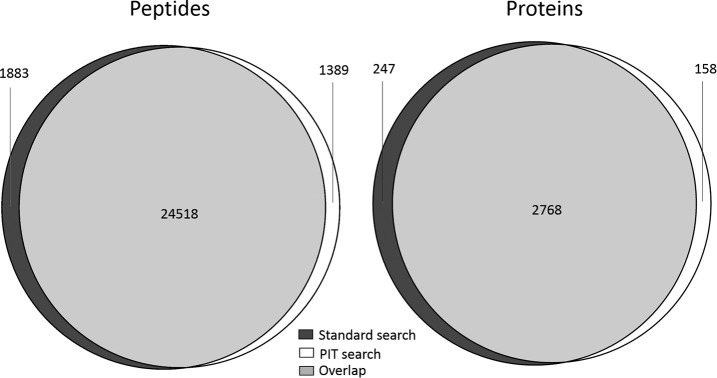
**Comparison of peptides and protein groups identified by a standard protein identification workflow in which mass spectra were searched against Uniprot, and a PIT workflow without reference genome, for HeLa cells infected with adenovirus.**

Comparing the PIT results against those obtained using the standard workflow, there is an overlap of 88% at the peptide level (taking into account only exact peptide sequence matches). A visual representation of these results is shown in Fig. 2. Again, these results are comparable with those obtained when PIT analysis was carried out previously on this data using other tools. Table II shows more detailed information about the number of peptides searched against in each case, and the peptide identifications that were unique to each search. This shows that the majority of peptides that were only found in one search were because of the absence of the peptide from the database used for the other search. The remaining nonoverlapping peptide identifications were because of other differences in the protein database, including the presence of sample-specific peptides in the PIT database that get matched to spectra that would otherwise be matched to peptides from Uniprot, and *vice versa*. It should also be noted that the peptide identifications were counted after protein grouping, which also affects which peptide identifications are retained.

**Table II:** Summary of search-specific peptide identifications and the proportion due to absence in the opposing database

Method	Peptides derived from database	Peptide identifications unique to this search	Unique identifications due to absence of peptide in other database
Standard (human and adenovirus)	11,636,910	1,883	1,878 (99.7%)
PIT–*de novo* assembly	7,843,921	1,389	813 (58.5%)
Standard (human only)	11,627,809	5,215	5,187 (99.4%)
PIT–genome guided assembly	7,095,081	846	552 (65.2%)

Overlap at the protein level is more difficult to define, partly because proteins are longer so the chance of an exact match between a sample-specific ORF and a Uniprot protein are lower than they would be at the peptide level, but also because peptides are assigned to protein ambiguity groups (PAGs) rather than distinct proteins. A PAG contains one or more proteins that are supported by a shared set of peptide evidence, with the protein with the most evidence being designated as an anchor protein that represents the PAG. Often several members of a PAG have equal peptide evidence, *e.g.* they may be isoforms, so the anchor is selected using some other criteria (*e.g.* in alphabetical order of their ID in the case of mzIdentML-lib's ProteoGrouper). Thus, when comparing lists of protein identifications from searches against different databases there are several reasons why anchor proteins may not match, even if the identified proteins are essentially the same. For the purposes of this comparison, a BLAST e-value below 1 × 10^−30^ (widely regarded as indicating strong homology between sequences) was taken to indicate a match between identified sequences. Using this threshold, a total of 2768 matches were found between PAGs from the standard protein identification workflow and PAGs from the PIT workflow (1955 anchor–anchor matches plus a further 813 matches between anchors and PAG submembers).

No match could be found between 247 of the PAGs from the standard workflow and the list of PAGs from the PIT workflow, *i.e.* 247 proteins were unique to the Uniprot search. Further analysis of these proteins was performed outside of GIO to understand why they were missed by the PIT search. Fourteen of the proteins were found to be isoforms or submembers of the overlapping PAGs. No transcript evidence could be found for the genes coding for 85 of the proteins (2.8% of the total identified by the standard workflow)—clearly PIT alone is unable to detect such proteins because the protein database is created purely from transcripts. Most of these proteins (71 out of 85), were also missing from the genome guided PIT workflow, suggesting either that the genes were not being expressed in the sample (possible because proteins can persist in a cell longer than the mRNA that was used to create them) or the RNA-seq depth was too shallow to detect them. The remaining 14 missing proteins can be attributed to Trinity's failure to assemble a small number of transcripts. Transcript evidence could be found for the remaining 148 proteins, but the ORF finding tool had either failed to produce ORFs from these transcripts or had produced ORFs that were not sufficient matches to proteins—this suggests an area for improvement in this aspect of the PIT methodology.

A total of 158 proteins were only found in the PIT search. On further inspection, 139 of these were found to match to the Uniprot proteome but were not found by the standard search, mostly because each protein only had single peptide evidence in that search, whereas two or more peptides were found by PIT. This often occurred because of a longer ORF sequence compared with the Uniprot protein, where the extra peptide matched to the extended region, suggesting potential deficiencies in the UniProt reference proteome that was used. The remaining 19 identified ORFs did not have significant homology to any known protein so have potential for future investigation as novel translated genomic elements—these ORFs, and the evidence for them, can be found in the supplementary data.

##### PIT Workflow for Genome Annotation

The genome annotating workflow was successfully applied to annotate the human genome with transcripts and peptides identified from the acquired RNA-seq and proteomic MS data. An example section of the GFF3 file visualized in the Genoverse (23) genome browser is shown in supplemental Fig. S4. Obviously the GFF3 file does not provide a complete annotation of the human genome, as only genes and proteins expressed in the individual analyzed sample are shown but it does allow for the rapid visualization of peptide evidence in a genomic context and the localization of putative novel translated genomic elements.

##### PIT Workflow with Reference Genome

Searching the acquired MS/MS data against ORFs derived from transcripts assembled by Cufflinks and TopHat using reference genome hg38, 21,601 peptides were found, which mapped to 2713 PAGs, whereas the standard workflow identified 2986 PAGs when only human proteins were used. Using the homology search tool included in this workflow, 2676 of these ORFs were identified as human proteins. Because this workflow used mapping to a human reference, adenovirus proteins were not considered.

**Fig. 3.**
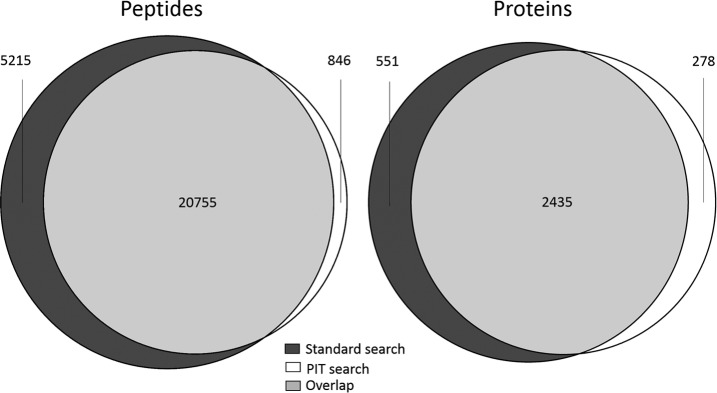
**Comparison of peptides and protein groups identified by a standard protein identification workflow in which mass spectra were searched against Uniprot, and a PIT workflow using a human reference genome, for HeLa cells infected with adenovirus.** Because a human reference genome was used, only human protein identifications are included in these results.

Comparing the PIT results against those obtained using the standard workflow, there is an overlap of 77% at the peptide level. A visual representation of these results is shown in Fig. 3.

Using the same PAG comparison method as in the previous section, a total of 2435 matches were found between PAGs from the standard protein identification workflow and PAGs from the PIT workflow (1630 anchor–anchor matches plus a further 805 matches between anchors and PAG submembers). The reduced overlap at both peptide and PAG level, when compared with the previous workflows, is mostly likely because Cufflinks produced fewer transcripts than Trinity (see Table I), resulting in a significantly smaller number of ORFs in the search database.

No match could be found between 551 of the PAGs from the standard workflow and the list of PAGs from the PIT workflow. However, isoforms of two of these proteins overlap with the PIT search. No transcript evidence could be found for 246 of these proteins. A total of 278 PAGs were only found in the PIT search. Of these, 241 ORFs had homology to human Uniprot proteins but were not identified by the standard search, mostly because these proteins had single peptide evidence in the standard search.

## DISCUSSION

Through the creation of GIO, we have demonstrated that Galaxy is an extremely effective platform for putting powerful integrative data analysis workflows into the hands of researchers. The PIT results obtained using GIO are very similar to those obtained previously from the same data using a bespoke hard-coded pipeline composed of different tools. The principal innovation is that the data analysis can now be reliably carried out by a typical bench biologist via a web interface without installing a single piece of software, whereas the original analysis required many months of programming and command line expertise. Although we have provided four reference workflows for PIT analysis, GIO permits huge flexibility in terms of generation of bespoke workflows for different applications. For example, users can experiment with different parameters, different tools for certain steps (*e.g.* peptide spectrum matching, post processing), or generate their own hybrid protein databases to search against using file manipulation tools that are already built in to Galaxy. Advanced users who install Galaxy on their own server are able to customize workflows even further, adding their application-specific downstream analysis tools for example.

In future, we plan to add further tools and workflows to GIO, to bring other complex proteomics data processing tasks within reach of the typical bench biologist. Priorities include tools for interpreting the results of PIT experiments, *e.g.* to permit fine grained comparison of mzIdentML files from different searches and to classify potentially novel translated genomic elements.

## Supplementary Material

Supplemental Data

## Footnotes

^1^ The abbreviations used are:

LC-MS/MS: Liquid chromatography tandem mass spectrometry
CID: Collision induced dissociation
FDR: False discovery rate
GIO: Galaxy Integrated Omics
ORF: Open reading frame
PAG: Protein ambiguity group
PIT: Proteomics informed by transcriptomics
RNA-seq: RNA sequencing
SILAC: Stable isotope labelling of amino acids in cell culture
TSV: Tab separated values.

## References

[1] Hubbard S. J. (2010) Computational approaches to peptide identification via tandem MS. Methods Mol. Biol. 604, 23–42 20013362 10.1007/978-1-60761-444-9_3

[2] Wilhelm M., Schlegl J., Hahne H., Moghaddas, Gholami A., Lieberenz M., Savitski M. M., Ziegler E., Butzmann L., Gessulat S., Marx H., Mathieson T., Lemeer S., Schnatbaum K., Reimer U., Wenschuh H., Mollenhauer M., Slotta-Huspenina J., Boese J. H., Bantscheff M., Gerstmair A., Faerber F., and Kuster B. (2014) Mass-spectrometry-based draft of the human proteome. Nature 509, 582–587 24870543 10.1038/nature13319

[3] Kim M. S., Pinto S. M., Getnet D., Nirujogi R. S., Manda S. S., Chaerkady R., Madugundu A. K., Kelkar D. S., Isserlin R., Jain S., Thomas J. K., Muthusamy B., Leal-Rojas P., Kumar P., Sahasrabuddhe N. A., Balakrishnan L., Advani J., George B., Renuse S., Selvan L. D., Patil A. H., Nanjappa V., Radhakrishnan A., Prasad S., Subbannayya T., Raju R., Kumar M., Sreenivasamurthy S. K., Marimuthu A., Sathe G. J., Chavan S., Datta K. K., Subbannayya Y., Sahu A., Yelamanchi S. D., Jayaram S., Rajagopalan P., Sharma J., Murthy K. R., Syed N., Goel R., Khan A. A., Ahmad S., Dey G., Mudgal K., Chatterjee A., Huang T. C., Zhong J., Wu X., Shaw P. G., Freed D. 6., Zahari M. S., Mukherjee K. K., Shankar S., Mahadevan A., Lam H., Mitchell C. J., Shankar S. K., Satishchandra P., Schroeder J. T., Sirdeshmukh R., Maitra A., Leach S. D., Drake C. G., Halushka M. K., Prasad T. S., Hruban R. H., Kerr C. L., Bader G. D., Iacobuzio-Donahue C. A., and Gowda-Pandey A. (2014) A draft map of the human proteome. Nature 509, 575–581 24870542 10.1038/nature13302 PMC4403737

[4] Hettich R. L., Pan C., Chourey K., and Giannone R. J. (2013) Metaproteomics: Harnessing the power of high performance mass spectrometry to identify the suite of proteins that control metabolic activities in microbial communities. Anal. Chem. 85, 4203–4214 23469896 10.1021/ac303053e PMC3696428

[5] Evans V. C., Barker G., Heesom K. J., Fan J., Bessant C., and Matthews D. A. (2012) De novo derivation of proteomes from transcriptomes for transcript and protein identification. Nat. Methods 9, 1207–1211 23142869 10.1038/nmeth.2227 PMC3581816

[6] Wang Z., Gerstein M., and Snyder M. (2009) RNA-Seq: a revolutionary tool for transcriptomics. Nat. Rev. Genet. 10, 57–63 19015660 10.1038/nrg2484 PMC2949280

[7] Goecks J., Nekrutenko A., Taylor J., and Galaxy Team. (2010) Galaxy: a comprehensive approach for supporting accessible, reproducible, and transparent computational research in the life sciences. Genome Biol. 11, R86 20738864 10.1186/gb-2010-11-8-r86 PMC2945788

[8] Gonzalez-Galarza F. F., Lawless C., Hubbard S. J., Fan J., Bessant C., Hermjakob H., and Jones A. R. (2012) A critical appraisal of techniques, software packages, and standards for quantitative proteomic analysis. Omics 16, 431–442 22804616 10.1089/omi.2012.0022 PMC3437040

[9] Jagtap P. D., Johnson J. E., Onsongo G., Sadler F. W., Murray K., Wang Y., Shenykman G. M., Bandhakavi S., Smith L. M., and Griffin T. J. (2014) Flexible and accessible workflows for improved proteogenomic analysis using the galaxy framework. J. Proteome Res. 12, 5898–5908 25301683 10.1021/pr500812t PMC4261978

[10] HUPO Proteomics Standards Initiative. www.psidev.info

[11] Ghali F., Krishna R., Lukasse P., Martinez-Bartolome S., Reisinger F., Hemjakob H., Vizcaino J. A., and Jones A. R. (2013) Tools (Viewer, Library and Validator) that facilitate use of the peptide and protein identification standard format, termed mzIdentML. MCP 12, 3026–3035 23813117 10.1074/mcp.O113.029777 PMC3820921

[12] Jones A. R., Eisenacher M., Mayer G., Kohlbacher O., Siepen J., Hubbard S. J., Selley J. N., Searle B. C., Shofstahl J., Seymour S. L., Julian R., Binz P. A., Deutsch E. W., Hermjakob H., Reisinger F., Griss J., Vizcaíno J. A., Chambers M., Pizarro A. (2012). The mzIdentML Data Standard for Mass Spectrometry-Based Proteomics Results. Mol. Cell. Proteomics : MCP, 11(7), M111.014381. doi:10.1074/mcp.M111.014381 PMC3394945 22375074

[13] Martens L., Chambers M., Sturm M., Kessner D., Levander F., Shofstahl J., Tang W. H., Römpp A., Neumann S., Pizarro A. D., Montecchi-Palazzi L., Tasman N., Coleman M., Reisinger F., Souda P., Hermjakob H., Binz P. A., Deutsch E. W. (2011). mzML—a community standard for mass spectrometry data. Mol. Cell. Proteomics 10, R110.000133. doi:10.1074/mcp.R110.000133 20716697 PMC3013463

[14] Kessner D., Chambers M., Burke R., Agus D., Mallick P. (2008) ProteoWizard: open source software for rapid proteomics tools development. Bioinformatics 24, 2534–2536 18606607 10.1093/bioinformatics/btn323 PMC2732273

[15] Galaxy Team, Galaxy Wiki. https://wiki.galaxyproject.org/Admin/Config/Pulsar

[16] Vaudel M., Barsnes H., Berven F. S., Sickmann A., and Martens L. (2011) SearchGUI: An open-source graphical user interface for simultaneous OMSSA and X!Tandem searches. Proteomics 11, 996–999 21337703 10.1002/pmic.201000595

[17] Kim S., and Pevzner P. A. (2014) MS-GF+ makes progress towards a universal database search tool for proteomics. Nat. Commun. 5, 5277 25358478 10.1038/ncomms6277 PMC5036525

[18] Grabherr M. G., Haas B. J., Yassour M., Levin J. Z., Thompson D. A., Amit I., Adiconis X., Fan L., Raychowdhury R., Zeng Q., Chen Z., Mauceli E., Hacohen N., Gnirke A., Rhind N., di Palma F., Birren B. W., Nusbaum C., Lindblad-Toh K., Friedman N., and Regev A. (2011) Full-length transcriptome assembly from RNA-Seq data without a reference genome. Nat. Biotechnol. 29, 644–652 21572440 10.1038/nbt.1883 PMC3571712

[19] Wu T. D., and Watanabe C. K. (2005) GMAP: a genomic mapping and alignment program for mRNA and EST sequences. Bioinformatics 21, 1859–1875 15728110 10.1093/bioinformatics/bti310

[20] Trapnell C., Pachter L., and Salzberg S. L. (2009) TopHat: discovering splice junctions with RNA-Seq. Bioinformatics 25, 1105–1111 19289445 10.1093/bioinformatics/btp120 PMC2672628

[21] Trapnell C., Williams B. A., Pertea G., Mortazavi A., Kwan G., van Baren M. J., Salzberg S. L., Wold B. J., and Pachter L. (2010) Transcript assembly and quantification by RNA-Seq reveals unannotated transcripts and isoform switching during cell differentiation. Nat. Biotechnol. 28, 511–515 20436464 10.1038/nbt.1621 PMC3146043

[22] Cox J., Neuhauser N., Michalski A., Scheltema R. A., Olsen J. V., and Mann M. (2011) Andromeda: a peptide search engine integrated into the MaxQuant environment. J Proteome Res. 10, 1794–1805 21254760 10.1021/pr101065j

[23] Genoverse team, Genoverse. www.genoverse.org

