# Supplementary material for: Galaxy Integrated Omics: Web-based Standards-Compliant Workflows for Proteomics Informed by Transcriptomics
Source: Mol Cell Proteomics. 2015 Aug 12;14(11):3087–93. doi: 10.1074/mcp.O115.048777 (PMC4638048; doi:10.1074/mcp.O115.048777)
Supplement: Supplemental Data [file supp_14_11_3087__index.html]

Galaxy Integrated Omics: Web-based standards-compliant workflows for proteomics informed by transcriptomics — Galaxy Integrated Omics: Web-based Standards-Compliant Workflows for Proteomics Informed by Transcriptomics — Galaxy Integrated Omics (GIO) — Supplemental Data 

# Galaxy Integrated Omics: Web-based Standards-Compliant Workflows for Proteomics Informed by Transcriptomics

## Supplemental Data

- Peptide and protein identifications - Peptide and protein identifications.
- Supplementary figures S1-S4 - Supplementary figures S1-S4.
