# Supplementary material for: Galaxy Integrated Omics: Web-based Standards-Compliant Workflows for Proteomics Informed by Transcriptomics
Source: Mol Cell Proteomics. 2015 Aug 12;14(11):3087–93. doi: 10.1074/mcp.O115.048777 (PMC4638048; doi:10.1074/mcp.O115.048777)
Supplement: Supplemental Data [file supp_O115.048777_mcp.O115.048777-2.pdf]

Galaxy Integrated Omics: Web-based standards-compliant workflows for proteomics informed by transcriptomics

Jun Fan, Shyamasree Saha, Gary Barker, Kate J Heesom, Fawaz Ghali, Andrew R Jones, David A Matthews, Conrad Bessant

Supplementary Figures

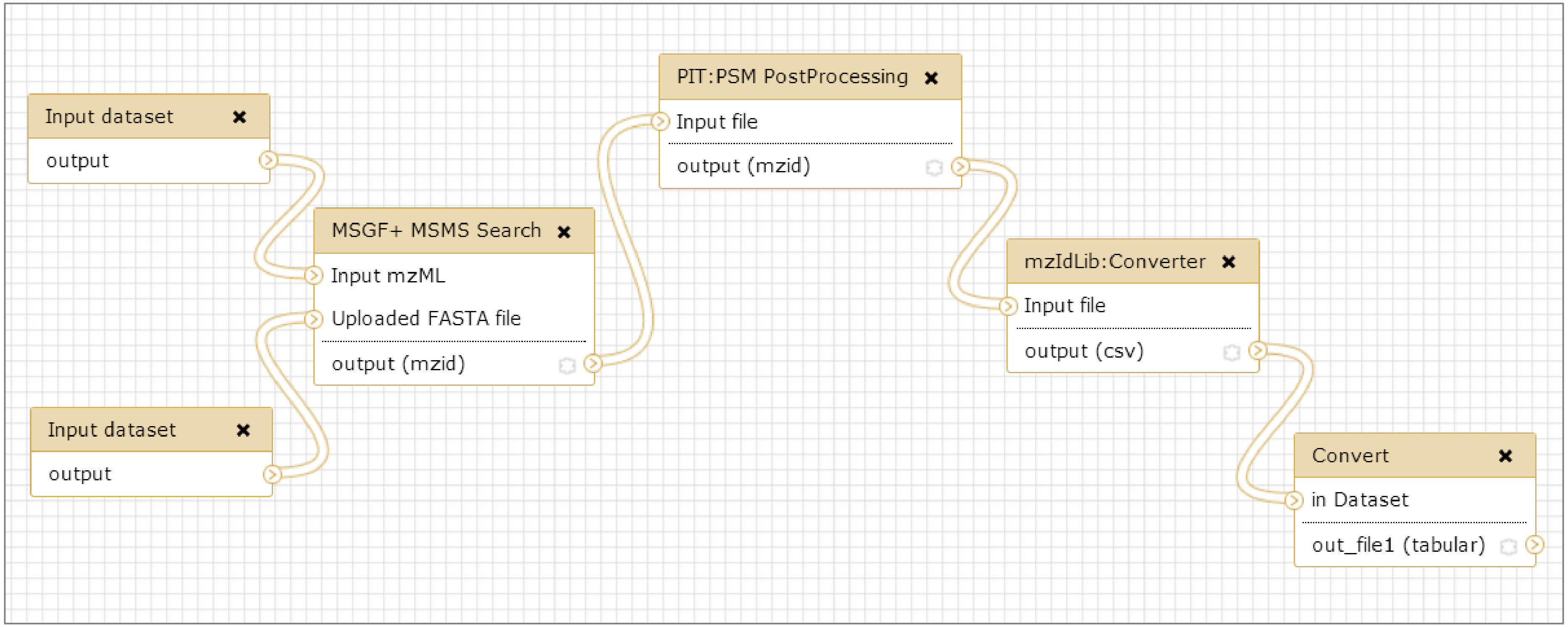

Figure S1: *Standard identification workflow. Inputs are proteomic MS data in mzML format, and a proteome to search against in FASTA format. The first step of the workflow is peptide spectrum matching, performed by MS-GF+. Post-processing is then performed to refine the list of PSMs and infer proteins from these. The output of these steps is a single mzIdentML file, from which the protein identifications are extracted and converted into a tabular (tab separated values - TSV) file for convenient viewing or downloading via the Galaxy interface.*

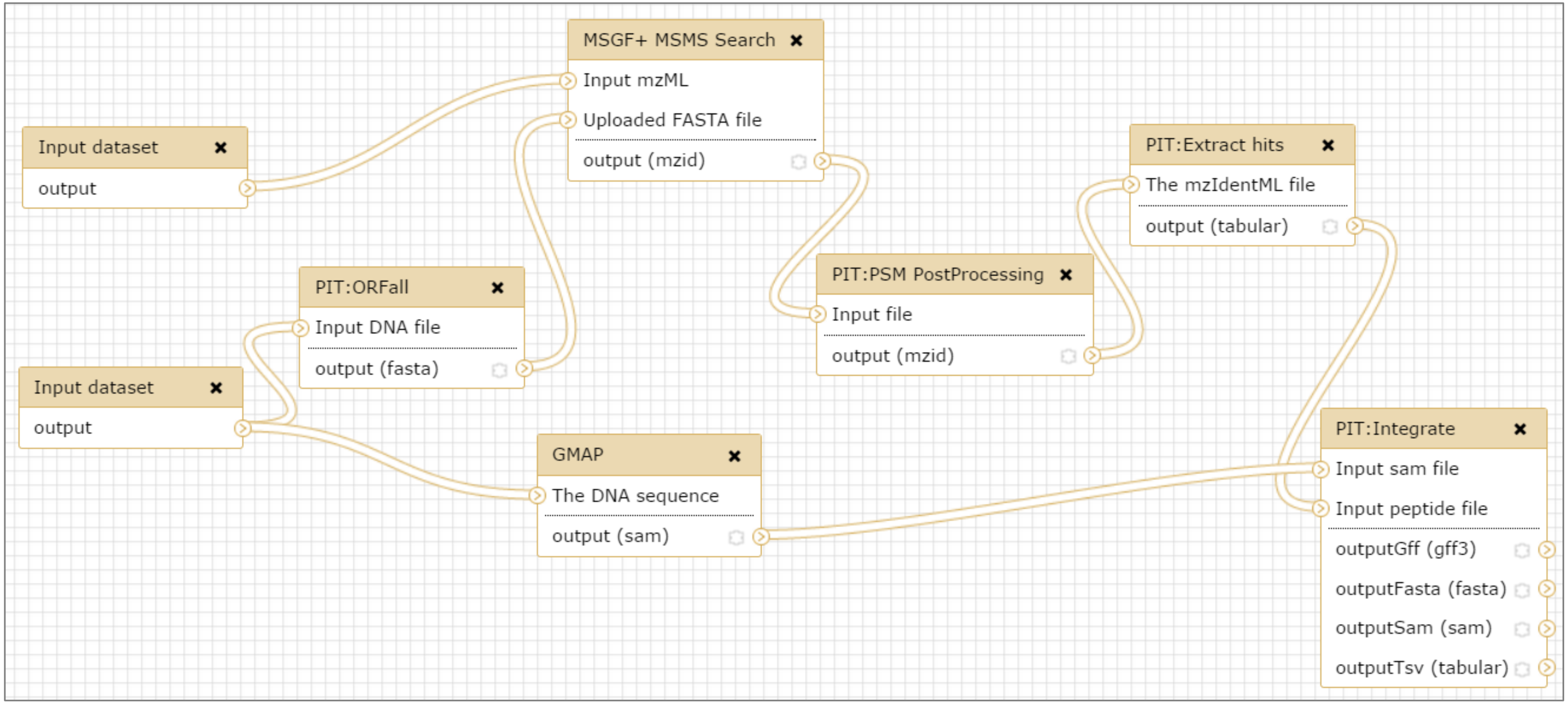

Figure S2: *Basic genome annotating proteomics workflow. The inputs to the workflow are a file containing raw spectral data and a list of transcripts assembled from RNA-seq data (assembled in a separate workflow using Trinity). In the first step of analysis, the longest open reading frames (ORFs) in all six frames for each transcript are determined using the in-house ORFall tool. MS-GF+ then finds peptide spectrum matches, which are post processed and grouped to proteins using tools from mzIdentML-lib. This results in a list of confidently identified ORFs. Alongside the peptide and protein identification,* de novo *assembled transcripts from the input file are mapped to a genome using GMAP. At the end of the workflow the in-house PIT:Integrate tool is used to integrate the identified transcriptomic and proteomic features into a single GFF3 genome annotation file so that these features can be viewed in their genomic context using a genome browser (see Figure S4).*

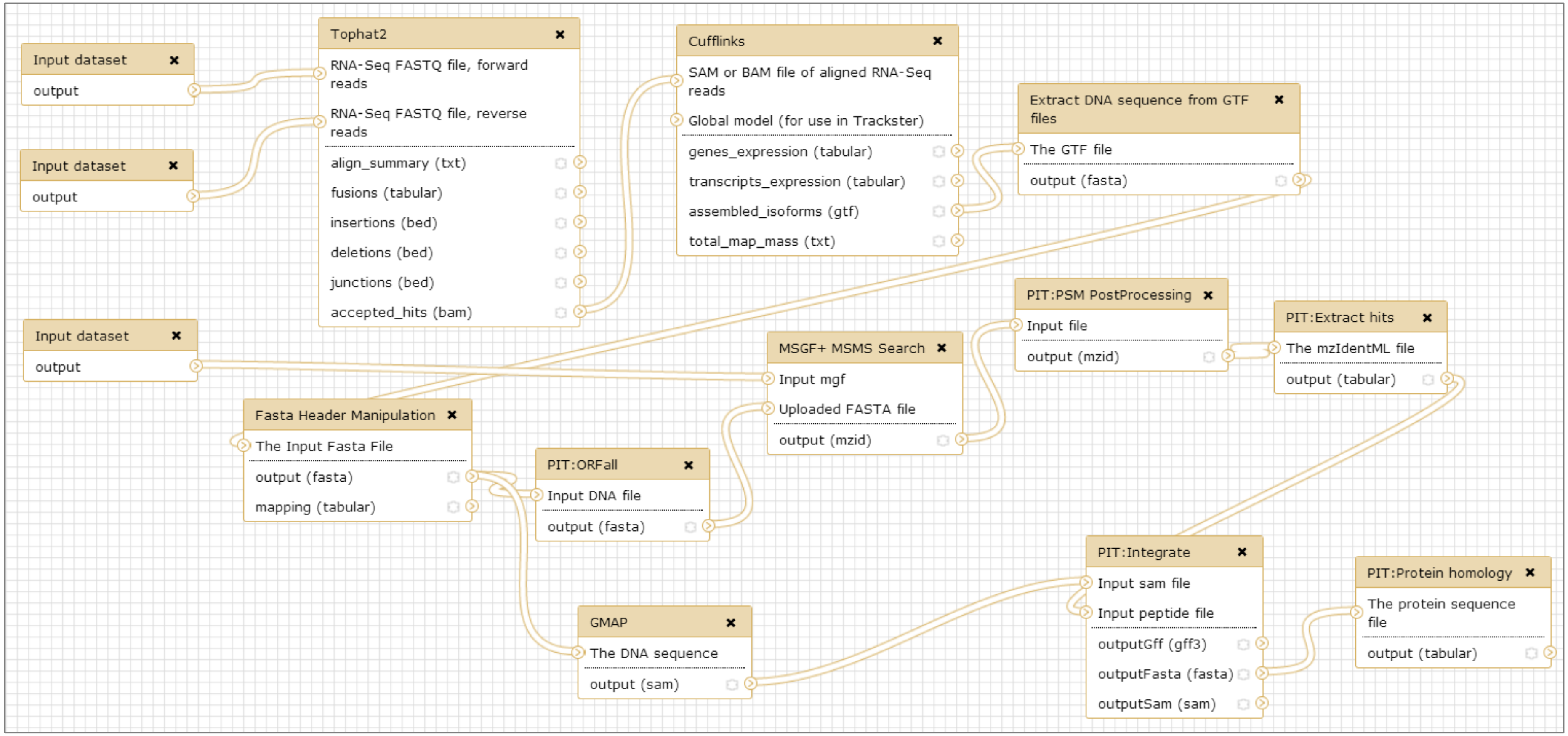

Figure S3: *PIT with reference genome. RNA-seq FASTQ files containing quality controlled forward and reverse reads are provided as inputs to TopHat2, which maps short reads to the genome of the species under study. This produces a BAM mapping file which is passed to Cufflinks to assemble the reads into transcripts. These transcripts are then used, as in the other workflows, to generate ORFs against which the acquired MS spectra are searched. Downstream processing also follows the same process as described previously for the other workflows.*

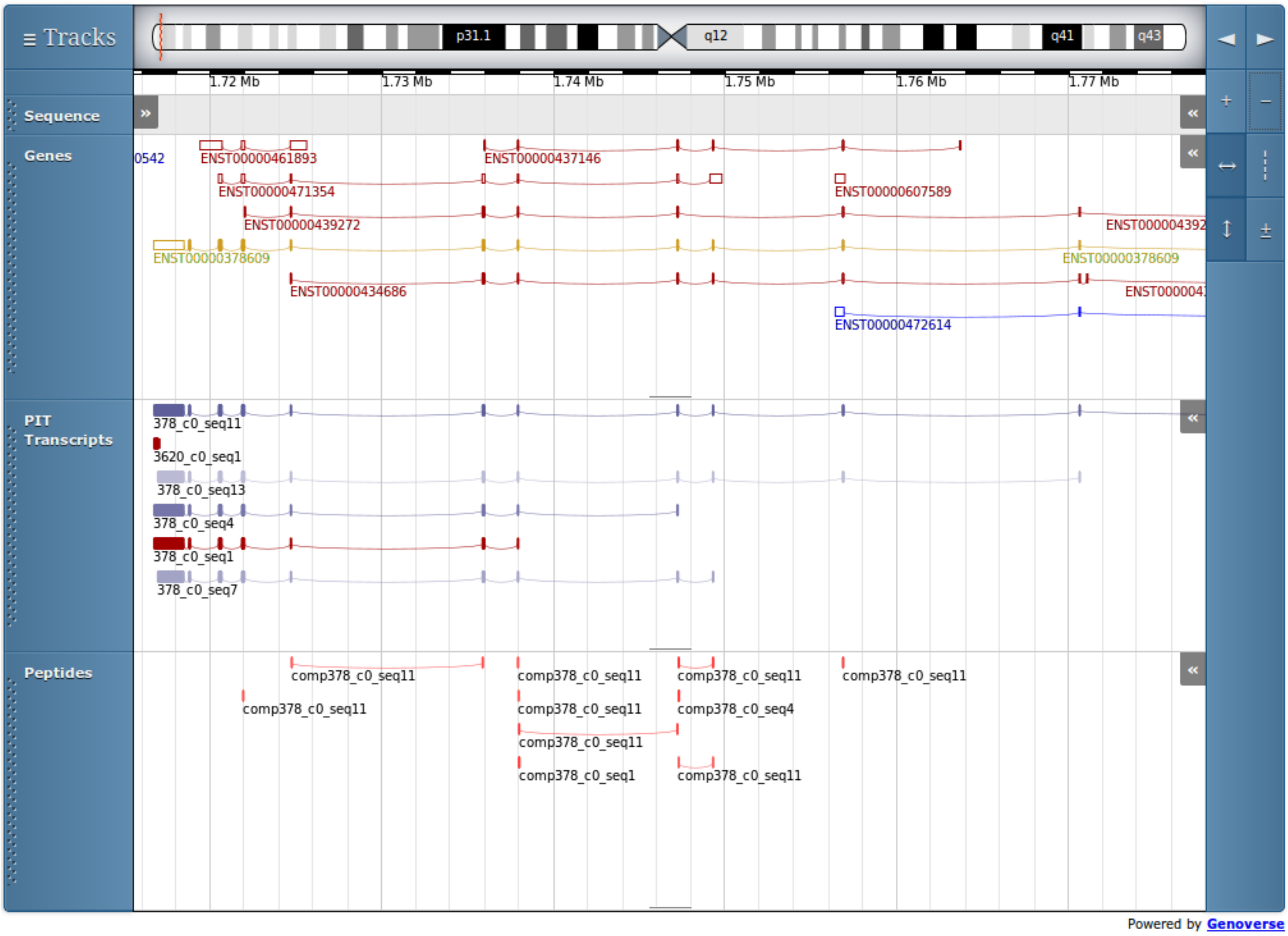

Figure S4: *Example of Genoverse view of a region of the human genome annotated with features from the GFF3 file produced by applying the genome annotating PIT workflow to RNA-seq and proteomic mass spectrometry data from adenovirus infected HeLa cells. The top two tracks give an indication of the region of the genome that is being show (in this case a region near the beginning of chromosome 1) and is used as a reference for the location of all the features in the lower tracks. The Genes track indicates the previously annotated gene structure from Ensembl transcripts (www.ensembl.org) – solid blocks indicate exons and the slightly curved horizontal lines between represent introns. The PIT Transcripts track shows transcripts assembled* de novo *from the acquired RNA-seq data, and the Peptides track shows peptide identifications derived from the mass spectrometry data. Genoverse was specially adapted to display intron-crossing peptides – a feature that is not readily available in other genome browsers.*
